# Alteration of somatosensory response in adulthood by early life stress

**DOI:** 10.3389/fnmol.2015.00015

**Published:** 2015-05-19

**Authors:** Yusuke Takatsuru, Noriyuki Koibuchi

**Affiliations:** Department of Integrative Physiology, Graduate School of Medicine, Gunma University, MaebashiJapan

**Keywords:** maternal deprivation, *in vivo* imaging, *in vivo* microdialysis, glutamate reseptor, spine

## Abstract

Early life stress is well-known as a critical risk factor for mental and cognitive disorders in adulthood. Such disorders are accompanied by altered neuro- (synapto-) genesis and gene expression. Because psychosomatic disorders induced by early life stress (e.g., physical and/or sexual abuse, and neglect) have become a socio-economic problem, it is very important to clarify the mechanisms underlying these changes. However, despite of intensive clinical and animal studies, such mechanisms have not yet been clarified. Although the disturbance of glucocorticoid and glutamate homeostasis by stress has been well-documented, it has not yet been clarified whether such disturbance by early life stress persists for life. Furthermore, since previous studies have focused on the detection of changes in specific brain regions, such as the hippocampus and prefrontal cortex, it has not been clarified whether early life stress induced changes in the sensory/motor system. Thus, in this review, we introduce recent studies on functional/structural changes in the somatosensory cortex induced by early life stress. We believe that this review provides new insights into the functional alteration of the somatosensory system induced by early life stress. Such information may have clinical relevance in terms of providing effective therapeutic interventions to early life stressed individuals.

## Introduction

Early life stress during the perinatal period induces functional and anatomical changes in the brain. Unfortunately, some of such changes persist in adulthood. Clinical studies have shown that early life stress during childhood persistently impairs cognitive and emotional functions, sometimes until adulthood (Chugani et al., 2001; Nelson et al., 2007; Gatt et al., 2009; Mueller et al., 2010; Gershon et al., 2013; Pesonen et al., 2013). Stress-induced alterations of neuronal activity and stress-related hormone secretion may affect neurological development such as dendrite arborization, synaptogenesis, and spine formation (McEwen, 1999; Vyas et al., 2002; Liston et al., 2006). These perinatal stress-induced morphological changes may alter the brain function throughout life (Romeo and McEwen, 2006; Shair, 2007; Gershon et al., 2013). However, it has not yet been clarified how the type, intensity and duration of stress affect different brain regions with different persistence (Romeo and McEwen, 2006). For example, although it has been well-known since several decades ago that acute stress disturbs glutamate and/or corticosterone homeostasis (Moghaddam, 1993; Moghaddam et al., 1994), it has not yet been clarified whether such a glutamate/corticosterone disturbance persists for a long time in a region- and temporal-specific manner after early life stress exposure (Popoli et al., 2011).

To study the effect of early life stress, various animal models have been introduced with their potential applications in humans (Shair, 2007). In these models, early life stress induces various disorders in adulthood, e.g., an enhancement of anxiety-related behaviors (Wigger and Neumann, 1999; Parfitt et al., 2004; Slotten et al., 2006; Shair, 2007). Such behavioral alteration is partly induced by structural changes of hippocampal neurons and changes in the rate of release of several neurotransmitters/hormones in this region (Brunson et al., 2005; Aisa et al., 2007; Oomen et al., 2010). The electrophysiological changes are also detected in the hippocampus of aged animals (Sousa et al., 2014). Early life stressed rodents also show neuronal changes such as those in synaptic spine density in the infralimbic cortex (Ovtscharoff and Braun, 2001). Early life stress also affects the expression levels of α-amino-3-hydroxy-5-methylisoxazole-4-propionic acid (AMPA) receptor by suppressing the function of Ca^2+^ calcium/calmodulin-dependent protein kinase type II (CaMKII) in the barrel cortex (Miyazaki et al., 2012). These findings indicate that early life stress during the perinatal period affects the brain function/structure in various brain regions. However, most studies have been performed focusing in several specific brain regions such as the hippocampus and prefrontal cortex (Wigger and Neumann, 1999; Parfitt et al., 2004; Slotten et al., 2006; Shair, 2007; Miyazaki et al., 2012). Thus, it has not yet been clarified whether early life stress disrupts the somatosensory function.

In this review, we discuss the effect of early life stress on somatosensory function by introducing our recent studies. Most of the results were obtained by *in vivo* studies such as *in vivo* imaging using two- (multi-) photon laser microscopy and free-moving *in vivo* microdialysis. This review may provide novel insights into the functional alterations of the somatosensory system induced by early life stress. Such information may be useful in terms of providing effective therapeutic interventions to early life stressed individuals.

## Persistent Alteration of Synaptic Turnover in the Somatosensory Cortex in Early Life Stressed Mice

**FIGURE 1:**
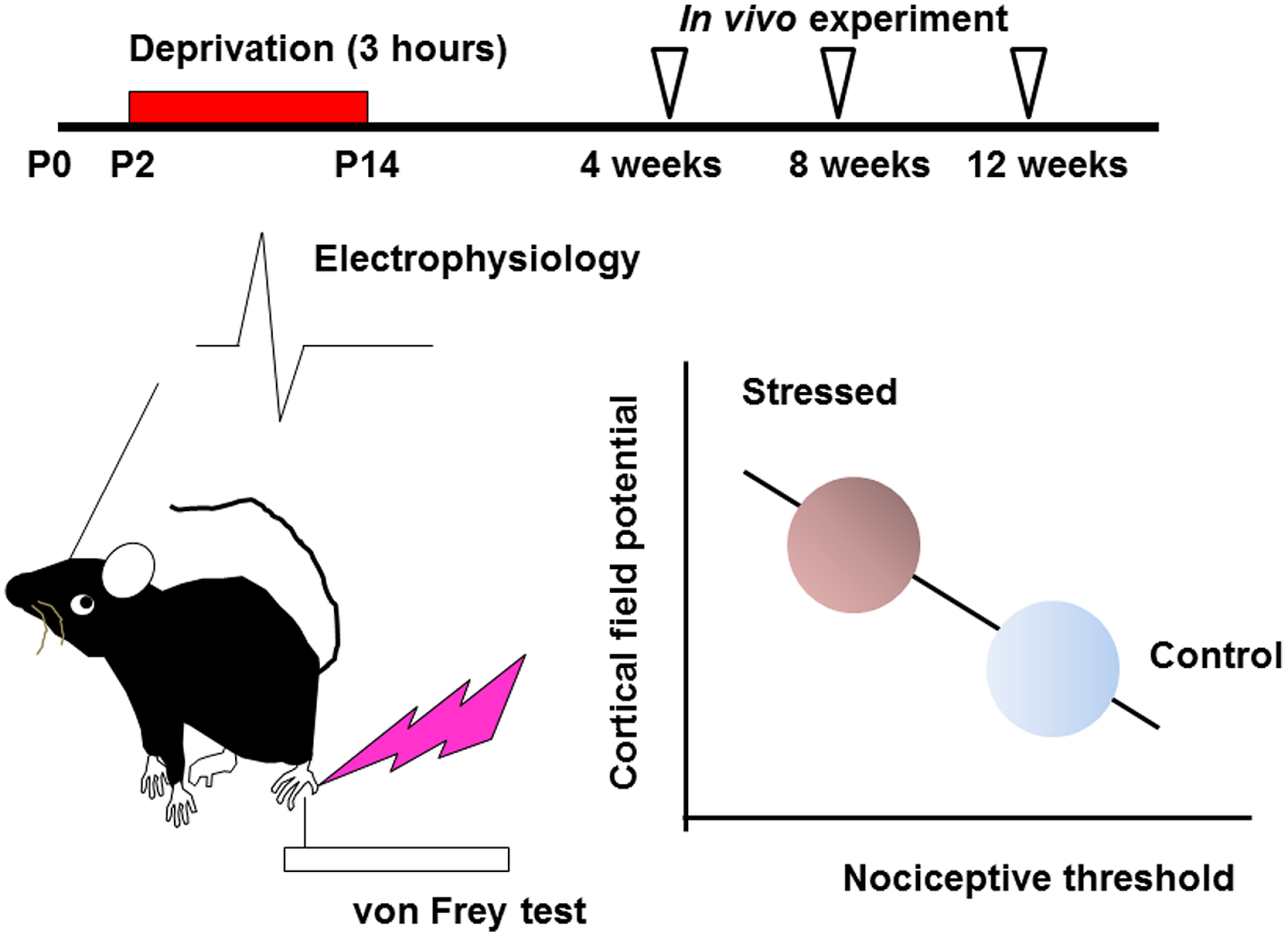
**Early life stress induces hyperactivity of somatosensory function (see Takatsuru et al., 2009a and Toya et al., 2014, for details)**. Maternal deprivation is performed from postnatal day P2 to P14, 3 h per day (the day of birth is determined as P0). After weaning, an *in vivo* experiment is performed from 4 to 12 weeks. In early life stressed mice, the nociceptive threshold studied by von Frey hair test decreases with increasing cortical field potential. These values significantly correlate.

Effects of early life stress can be seen in various brain regions. Such effects are observed as changes in synaptic spine density, synaptic turnover rate, electrophysiological properties, neurotransmitters release or expression levels of neuronal and glial proteins. However, it is difficult to identify specific regions affected by a specific early life stress. Even if a particular neuropsychological phenotype is observed, it may still be difficult to identify the affected brain regions, because of the complexity of the mechanisms underlying these changes. In the hippocampus, for example, early life stress alters the spine density and dendritic outgrowth of pyramidal neurons (Magarinos and McEwen, 1995; Pawlak et al., 2005; Monroy et al., 2010; Magarinos et al., 2011), with the change in the expression levels of proteins such as neurotrophic factors and transcription factors (Lippmann et al., 2007; Nair et al., 2007; Kawano et al., 2008; Magarinos et al., 2011; Horii-Hayashi et al., 2013; Suri et al., 2013; Dimatelis et al., 2014). Such an alteration may lead to impaired memory acquisition and cognitive function (Huot et al., 2002; Aisa et al., 2007; Fabricius et al., 2008; Suri et al., 2013; Connors et al., 2015). However, involvement of other brain region cannot be excluded. On the other hand, the somatosensory cortex receives sensory information such as pain, temperature, and pressure, integrates them to identify the object (Haggard, 2006). Its disorder produces difficulties in interpreting tactile information (Freund, 2003; Tinazzi et al., 2013). However, the effect of early life stress on somatosensory function has not yet been clarified. In the somatosensory cortex, the spine density is rather stable after early life stress compared with those in the hippocampus not only in juveniles but also in adults (Takatsuru et al., 2009a). Nevertheless, the nociceptive threshold is significantly decreased in early life stressed juvenile (4 weeks-old) and adult mice (12 weeks-old; Takatsuru et al., 2009a). Electrophysiologically, the slope (μV/ms) of field potential in layer II/III evoked by vibrotactile somatosensory simulation increases in early life stressed adult mice. Interestingly, these changes significantly correlate with the decrease in nociceptive threshold (**Figure 1**; Toya et al., 2014), indicating that somatosensory function is persistently altered by early life stress.

As discussed above, although the somatosensory response is altered by the change in electrophysiological properties, previous studies have shown slight morphological changes in the somatosensory cortex (Takatsuru et al., 2009a). To clarify further the mechanism inducing neurological alterations, it is necessary to apply additional techniques such as two-photon laser microscopy (Denk et al., 1990; Grutzendler et al., 2002; Trachtenberg et al., 2002). This technique enables us to examine dynamic changes in synaptic turnover rate and neuronal excitability during neuronal circuit remodeling (Takatsuru et al., 2009b).

In early life stressed mice, the turnover rate of mushroom spines, which usually maintain their structure for a long time (Grutzendler et al., 2002; Trachtenberg et al., 2002), is significantly increased in the somatosensory cortex in not only juvenile but also in adult mice (Takatsuru et al., 2009a). Because both of the gain and loss of spines occur simultaneously, the total number of spines is not markedly altered. However, two-photon microscopy enabled us to detect the persistent dynamic changes in synaptic turnover induced by early life stress. These findings indicate that early life stress destabilizes synaptic formation in the somatosensory cortex, resulting in the disturbance of somatosensory function.

Although we have shown the persistent increase in synaptic turnover rate in the somatosensory cortex induced by early life stress, the mechanisms underlying such an alteration have not yet been clarified. One possibility is the involvement of microglia. Recently, we have found the alteration of motility of microglia in early life stressed mice *in vivo* (Takatsuru et al., 2015). The motility of the filopodia-like processes is increased in early life stressed mice. Interestingly, the motility of the processes negatively correlates with the somatosensory threshold (the motility is higher in the mice with a lower threshold). Furthermore, the number of processes is significantly increased in early life stressed mice after acute somatosensory stimulation and such an increase persists for several hours. The motility of microglia is changed by neuronal conditions as in the case of remodeling of synapses after a focal stroke (Wake et al., 2013). Because previous studies indicate that microglia may partly regulate synaptic formation by removing or ‘stripping’ synapses (Marín-Teva et al., 2004; Cullheim and Thams, 2007; Tremblay, 2011) and because the direct contact of microglial processes with spines has been observed (Wake et al., 2013), the activity of microglia increased by early life stress may contribute to the structural instability of spines in the somatosensory cortex. In a series of study, we have clarified that not only severe brain damage such as ischemia or inflammation, but also psychological stress such as maternal separation can produce persistent changes in microglial activity. However, early life stress factors activating microglia have not yet been clarified. In the next section, we will discuss several possible factors for such activation.

## Persistent Alterations of Glutamate and Glucocorticoid Homeostasis in the Somatosensory Cortex Induced by Early Life Stress

**FIGURE 2:**
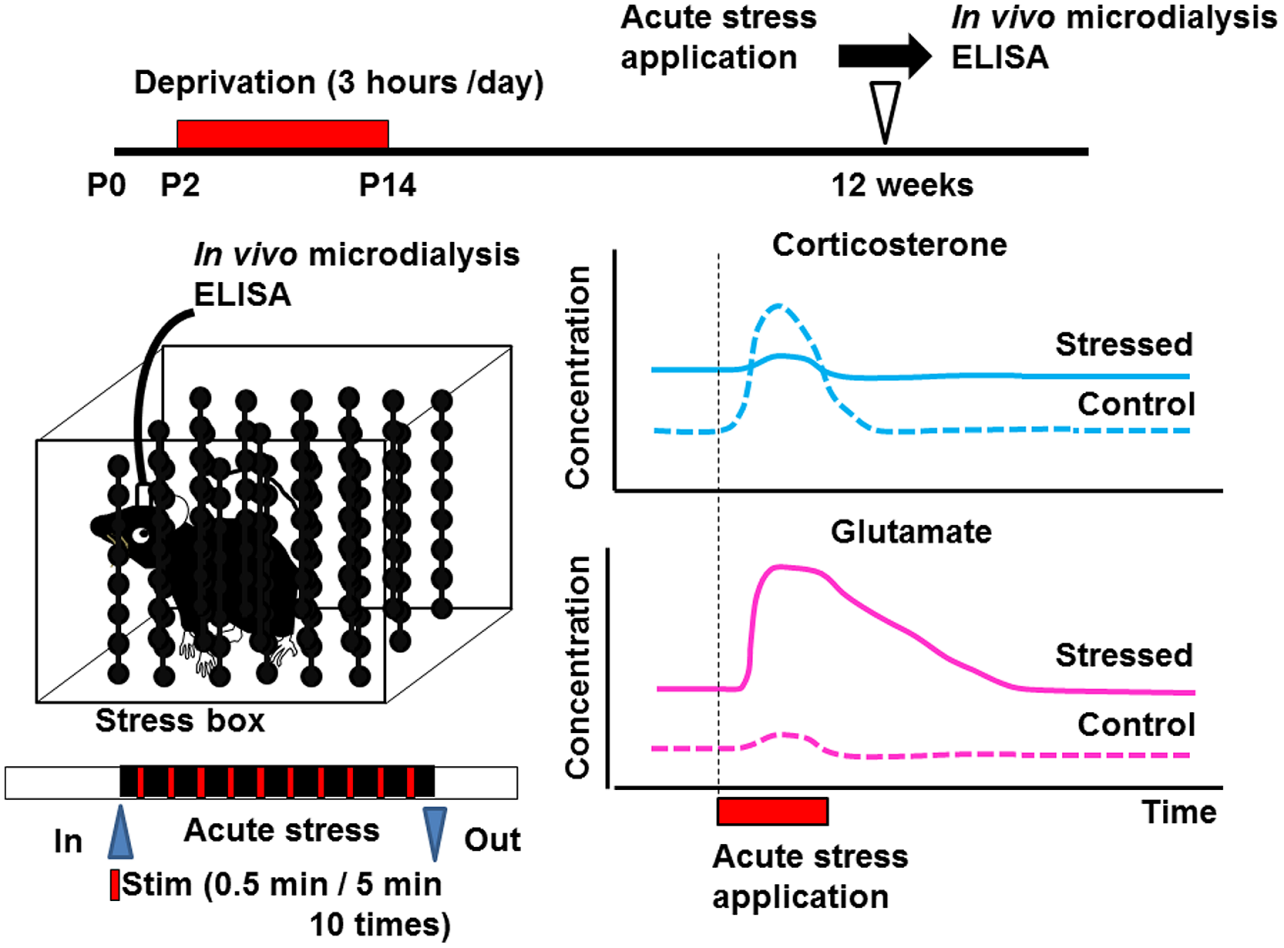
**Schematic drawing showing the effect of early life stress on acute-stress-induced corticosterone and glutamate responses in the somatosensory cortex**. Samples from the somatosensory cortex were collected by *in vivo* microdialysis. After collecting the dialysate in the home cage, the mice were placed inside the stress box. Stressful physical stimulation (30 s duration, every 5 min) were applied 10 times. The concentrations of corticosterone and glutamate were determined by ELISA and HPLC, respectively, (Toya et al., 2014). Early life stress induced an increase in basal levels of corticosterone and glutamate. On the other hand, although acute-stress-mediated increase in corticosterone concentration did not occur in early life stressed mice, that in glutamate concentration was markedly enhanced.

Early life stress may induce alterations of neurotransmitters (Barbosa Neto et al., 2012; Martisova et al., 2012; Gunn et al., 2013) and neurotrophic factors in various brain regions (Lippmann et al., 2007; Nair et al., 2007; Kawano et al., 2008; Magarinos et al., 2011; Horii-Hayashi et al., 2013; Suri et al., 2013; Dimatelis et al., 2014). Such alteration may produce changes in the structure of neural circuits, spine turnover rate, and/or microglial motility. Since many critical developmental events occur in the neonatal period, even weak environmental insults may produce irreversible alterations in organ homeostasis. Indeed, disruption of homeostasis of glucocorticoid release by administering excess amounts of corticosterone produces various abnormalities such as those in the number of spines, synaptic turnover rate, microglial motility, electrophysiological properties, and astrocyte function (Gunn et al., 2013). However, it has not yet been clarified whether such alterations occur in the somatosensory cortex. Detecting the concentration of a neurotransmitter such as glutamate or GABA in the cortex may reveal the mechanisms underlying the change in the turnover rate of mushroom spines. *In vivo* microdialysis is a potent technique to observe the neuronal transmitter release under intact condition although the spatial resolution is limited. This technique also enables local drug application, which is usually difficult due to the blood–brain barrier (Takatsuru et al., 2013). Using this technique, we have found that the concentration of glutamate is increased in early life stressed mice under free-moving condition (**Figure 2**; Toya et al., 2013, 2014). We have also reported that the homeostasis of coritcosterone is also affected by early life stress (Toya et al., 2014).

Corticosterone may play a protective role in neuronal circuits against stress (McEwen, 2000a,b). Glucocorticoids convert proteins and/or lipids into carbohydrates, which can be easily used for energy production. This conversion will serve the body well in the short run by replenishing energy reserves after a period of activity, as in a situation such as running away from a predator (McEwen, 2000b). Glucocorticoids also increase the appetite for food and promote food-seeking behavior (Leibowitz and Hoebel, 1997). Thus, enhancement of corticosterone release under acute stress potentially protects the body from stress. However, a long-term increase in corticosterone concentration induces dendritic atrophy in some brain regions (McEwen, 2000a) and thus, the concentration of corticosterone should be carefully controlled to maintain the homeostasis of body/brain functions. Furthermore, perinatal stress sometimes disrupts hypothalamic-pituitary-adrenal axis, resulting into persistent aberrant glucocorticoid secretion (Faturi et al., 2010; Koe et al., 2014; Nishi et al., 2014). Under such conditions, the protective role of glucocorticoids in neural circuits may be disrupted. Thus, an increased basal corticosterone concentration in the somatosensory cortex in early life stressed animals may be partly involved in inducing spine instability, glutamate secretion, and microglia motility. Disruption of glucocorticoid responses to acute stress may exacerbate such instability.

In early life stressed animals, the basal concentration of glutamate in the somatosensory cortex increases markedly (Toya et al., 2014). It has been well-known that the acute stress disrupts glutamate homeostasis (Moghaddam, 1993). However, it has not yet been clarified whether such disruption persists for life after early life stress in a specific brain region. Our study clearly demonstrated the increase in glutamate level in adulthood by early life stress in the somatosensory cortex. On the other hand, in control mice, the concentration of glutamate is rather stable after acute-stress application (Toya et al., 2014). This is probably due to the activation of glial cells that take up excess glutamate, thus preventing excitotoxity (Danbolt, 2001; Takayasu et al., 2009). In early life stressed mice, on the other hand, although the basal glutamate concentration is sixfold that in control mice, acute stress further increased the concentration of glutamate. Such a further increase lasts longer than 1 h after stimulation. These findings indicate the alteration of glutamate homeostasis by early life stress. However, the interaction between enhanced glutamate release and suppressed corticosterone response induced by acute stress early in life of mice has not yet been clarified.

Although the mechanisms inducing the persistently enhanced glutamate release in the somatosensory cortex induced by early life stress have not yet been fully understood, previous studies have provided several clues. Environmental stress enhances glutamate release and suppresses glial-cell-mediated glutamate cycling. Such changes affect synaptic transmission in the limbic/cortical areas (Sanacora et al., 2012). Early life stress also affects the structural organization, i.e., dendritic remodeling, reduction of synaptic spine formation, glial cell loss, and possibly volumetric reductions of several specific brain regions of the rodent brain (Sanacora et al., 2012). Acute exposure to stress or administration of glucocorticoids rapidly promotes glutamate release in the hippocampus and other brain regions (Lowy et al., 1993; Moghaddam, 1993; Reznikov et al., 2007). The glucocorticoid-receptor-mediated increase in the expression levels of presynaptic soluble *N*-ethylmaleimide-sensitive factor attachment protein receptor (SNARE) protein complexes are induced in the presynaptic membrane in the prefrontal/frontal cortex by acute stress. SNARE then enhances the release of glutamate (Musazzi et al., 2010). The Rab4-mediated recycling of NMDA and AMPA receptors from early endosomes is also enhanced in the prefrontal cortex (Yuen et al., 2009). Chronic stress also decreases the number of glial fibrillary acid protein (GFAP)-expressing cells and the impaired clearance of synaptic glutamate through excitatory amino acid transporters in the prefrontal cortex (Banasr and Duman, 2008). Taken together, it is reasonable to speculate that early life stress may alter glucocorticoid homeostasis, and such an alteration may enhance glutamate release by stimulating the expression of proteins related to glutamate release and sensitivity. The activity of glial cells, which take up glutamate, may also be affected, inducing an increase in interstitial glutamate level.

On the basis of the findings mentioned above, we examined the involvement of glutamate receptor subunits of AMPA, NMDA, and metabotropic glutamate receptors after application of acute stress in early life stressed mice to clarify the underling mechanisms by Western blot analysis (Toya et al., 2013). However, protein levels of these subunits in the membrane fraction were not significantly different between the control and early life stressed animals before and after acute-stress application. Because we carried out only Western blot analysis, further study to determine the protein turnover rate by *in vivo* imaging may be required. Under the present condition, however, we were unable to detect the involvement of glutamate receptors in the alteration of somatosensory function induced by early life stress.

## Summary and Perspectives

**FIGURE 3:**
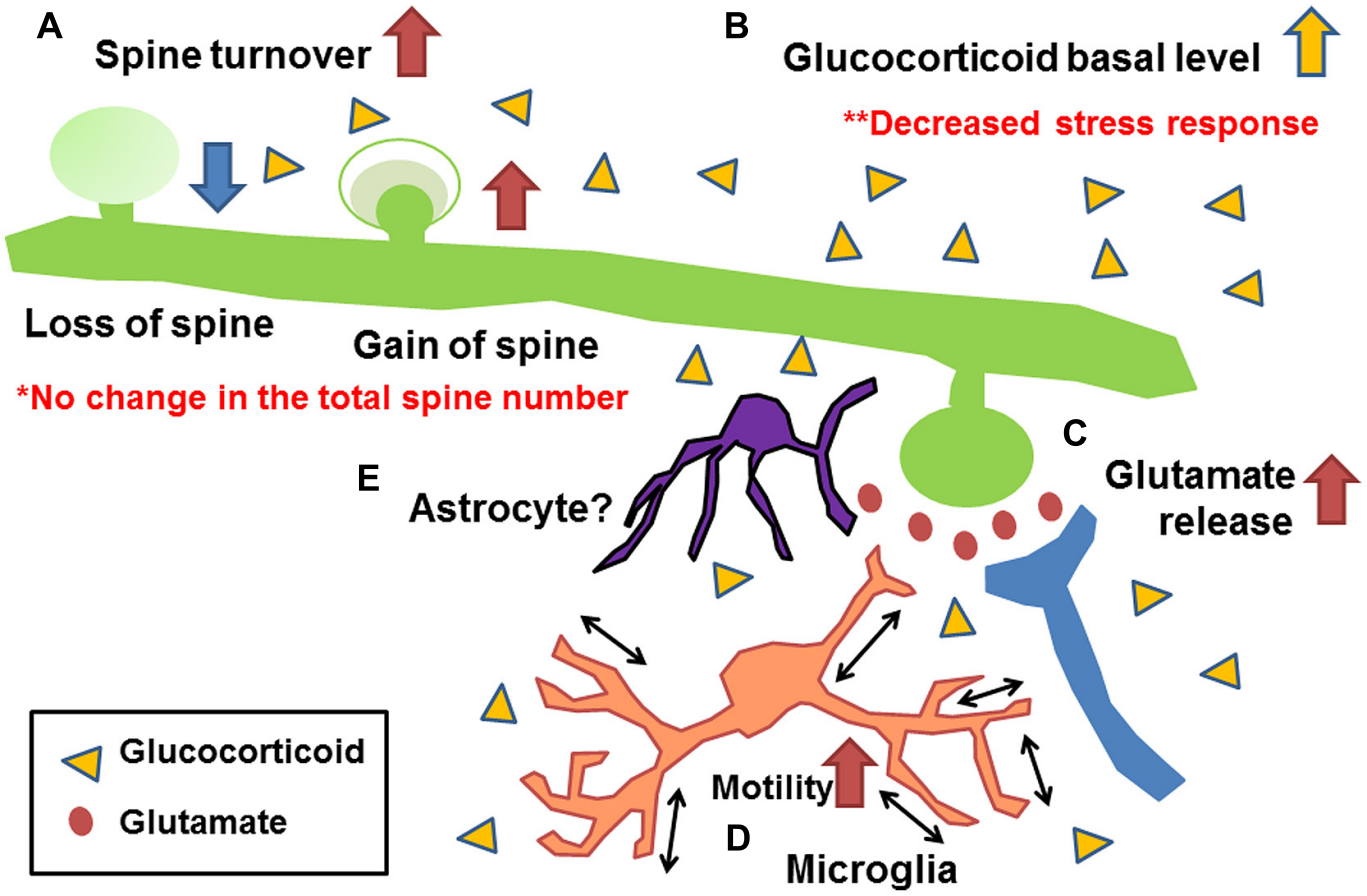
**Schematic drawing showing the morphological/chemical alterations induced by early life stress in the somatosensory cortex (Takatsuru et al., 2009a, 2015; Toya et al., 2014). (A)** Increase in mushroom spine turnover rate without significant alteration of total number of spines, **(B)** increase in basal glutamate level and acute-stress-mediated increase in glutamate level, **(C)** increase in basal level of corticosterone without further increase under acute stress, and **(D)** increase in microglial motility. Although the involvement of astrocytes has not yet been clarified **(E)**, studies of other brain regions indicate their possible involvement (Gunn et al., 2013).

**Figure 3** shows a summary of the effects of early life stress in the somatosensory cortex. We found the following; (A) Increase in mushroom spine turnover rate without significant alteration of the total number of spines, (B) Increase in basal level of corticosterone without further increase under acute stress, (C) Increase in basal and acute-stress-mediated glutamate levels, and (D) Increase in microglial motility. A combination of these alterations may have affected somatosensory function. Unfortunately, however, we failed to identify molecules involved in such alterations. One possible reason for the failure is that, although we detected a significant increase in synaptic turnover rate and microglial motility with a decreased somatosensory threshold and an increased electrophysiological activity, less than 10% of all spines were lost or gained. Thus, only a limited amount of molecules may be involved in such subtle changes. To detect such changes, Western blot analysis may not be a suitable technique. More sophisticated techniques such as detection of protein trafficking and/or protein expression at the single-cell level may be required. Nevertheless, our series of studies have demonstrated the persistent alteration of somatosensory function induced by early life stress with morphological/chemical alterations in the somatosensory cortex. Although the involvement of astrocytes has not yet been clarified, a previous study has shown that early life stress decreases glutamate uptake in the hypothalamus (Gunn et al., 2013). Thus, such a decrease may also be induced in the somatosensory cortex (**Figure 3E**). Trials to clarify the involvement of astrocytes are currently underway. However, as discussed above, the morphological/chemical changes in the somatosensory cortex are not always identical to those in other brain regions. Thus, more careful analysis may be required to examine the brain region-specific effects of early life stress. In particularly, more attention should be paid to the change in somatosensory function in early life stressed humans, because only limited information is available at present. We believe that this review has provided an important clue to developing effective therapeutic interventions to prevent persistent somatosensory abnormalities induced by early life stress.

## Conflict of Interest Statement

The authors declare that the research was conducted in the absence of any commercial or financial relationships that could be construed as a potential conflict of interest.
